# Hypoplastic Posterior Leaflet of the Tricuspid Valve—a Unique Cause of Right Heart Failure

**DOI:** 10.1016/j.case.2023.11.009

**Published:** 2024-03-08

**Authors:** Raja Ezman Raja Shariff, Mohd Rahal Yusoff, Khairul Shafiq Ibrahim, Sazzli Kasim

**Affiliations:** Universiti Teknologi Mara, Sungai Buloh, Selangor, Malaysia

**Keywords:** Tricuspid valve, Congenital heart disease, Hypoplastic tricuspid valve, Transesophageal echocardiography, Case report

## Abstract

• TR can be due to either primary or secondary causes.
• Primary TR due to congenital hypoplasia of leaflets is rare.
• Multimodality imaging is key in identifying the cause of TR.

## Graphical abstract

**Figure:**
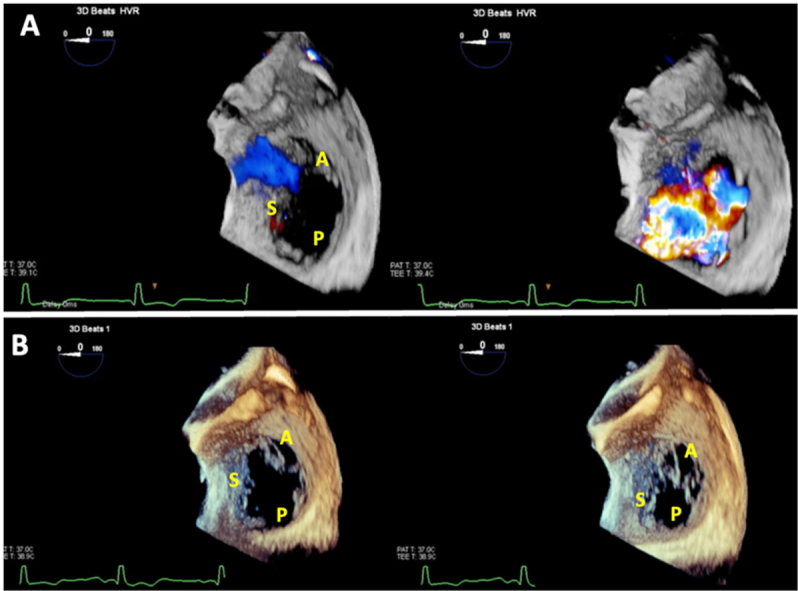

## Introduction

Tricuspid regurgitation (TR) can largely be divided into 2 categories—primary and secondary, the former being uncommon. Causes of primary TR are either congenital (e.g., Ebstein’s anomaly) or acquired (e.g., endocarditis, myxomatous disease, pacemaker lead-related, etc.).^1^ We report a patient with a rare finding of severe TR due to hypoplasia of the posterior leaflet of the tricuspid valve (TV) leading to delayed presentation for right heart failure.

## Case Presentation

**Figure 1:**
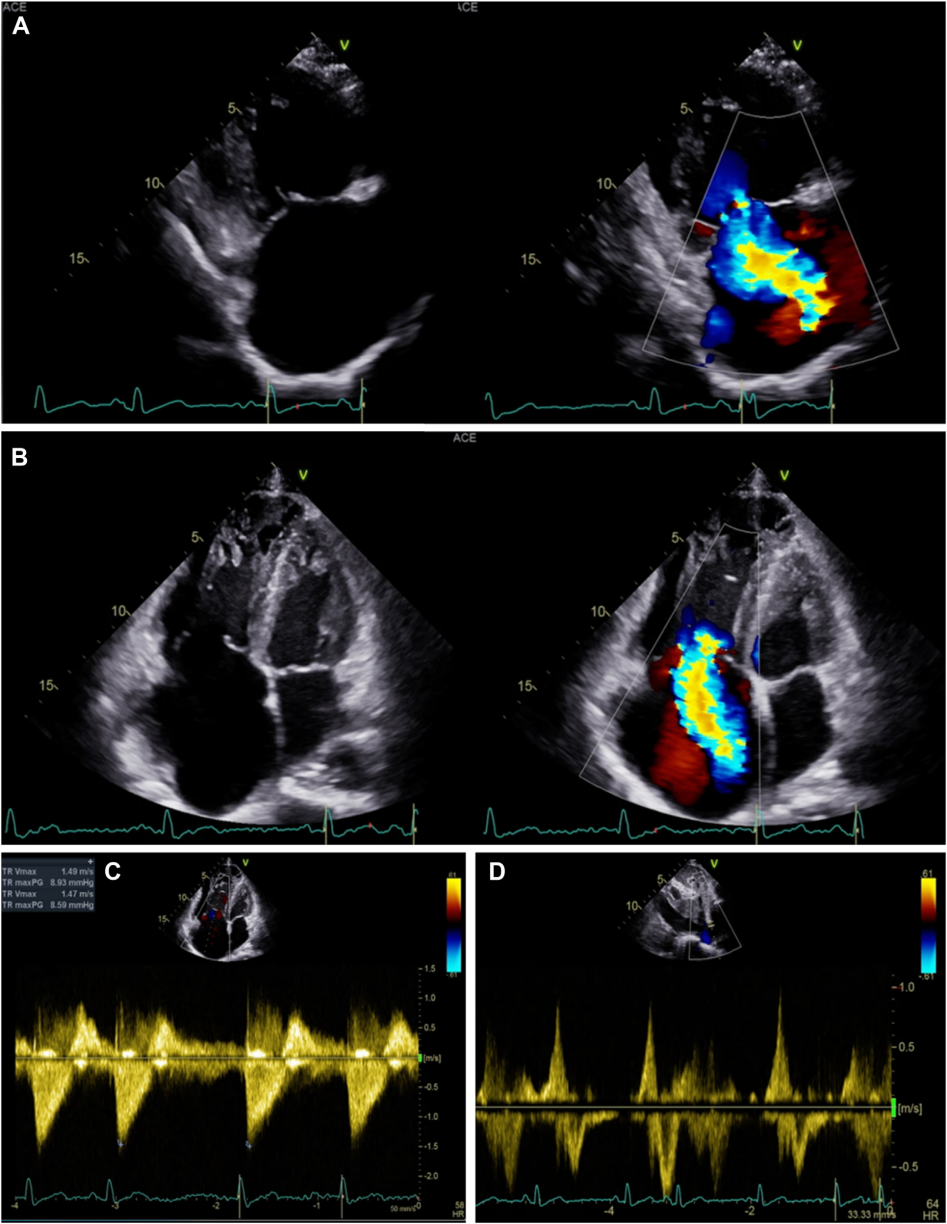
Two-dimensional TTE, RV inflow **(A)** and apical 4-chamber **(B)** views, without (*left*) and with (*right*) color-flow Doppler, demonstrates the dilated right heart and severe TR. Continuous-wave Doppler through the TV **(C)** demonstrates a triangular-shaped, low-velocity spectral Doppler pattern of the severe TR. Pulsed-wave Doppler within the superior hepatic vein **(D)** demonstrates systolic flow reversal.

**Figure 2:**
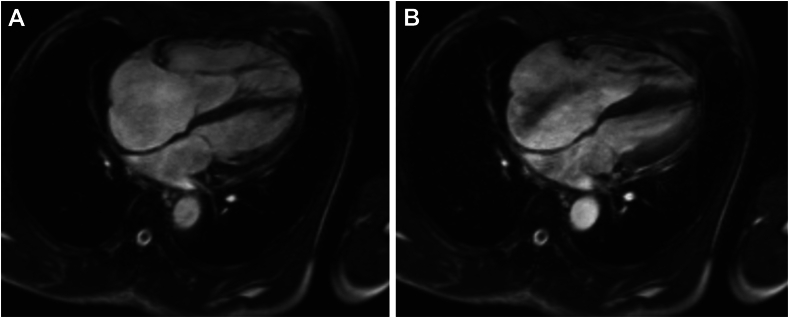
Cardiovascular magnetic resonance imaging, balanced steady-state free precession sequence, 4-chamber view in diastole **(A)** and systole **(B)**, demonstrates the dilated RA and signal loss from dephasing, suggesting severe TR.

A 57-year-old man was referred to our center with complaints of bilateral leg swelling and worsening dyspnea over an 18-month period for which the patient had never sought medical attention. The patient was not known to have any other illnesses, denied intravenous drug use, and worked as an office clerk. There was no history of any hospitalization in the past and no known history of risk factors linked to endocarditis. The chest radiograph from the referring center revealed cardiomegaly, and the electrocardiogram showed sinus rhythm with evidence of right ventricular (RV) strain. The clinical examination revealed a respiratory rate of 24 breaths/minute, oxygen saturations of 96% on room air, temperature of 36.4°C, raised jugular venous pressure, a right parasternal heave, and a loud second heart sound. There was also an audible, grade 4 pansystolic murmur, best heard at the right lower sternal border and loudest on inspiration. Examination of the lungs was unremarkable. A transthoracic echocardiogram (TTE) was performed in view of clinical findings, demonstrating both right atrial (RA) dilatation (RA volume index of 103.0 mL/m^2^) and RV dilatation (basal RV diameter of 5.5 cm, mid RV diameter of 5.1 cm, and proximal RV outflow tract diameter of 5.1 cm), with mildly reduced left ventricular function (Figure 1, Video 1, Video 2, Video 3, Video 4). The TV annulus measured largest at 5.0 cm in diameter. Using the RV inflow view with the coronary sinus in view (marking the most posterior aspect of the valve), the posterior leaflet of the TV was seen as being hypoplastic, leading to a large coaptation gap and severe TR. There was also evidence of tethering of the septal leaflet appreciated (Video 2). Color-flow Doppler demonstrated torrential TR (vena contracta width of 24 mm and effective regurgitation orifice area of 92.0 mm^2^). Despite peak TR velocity (TR Vmax) measuring only at 1.5 m/sec, there was a noticeable triangular continuous-wave Doppler signal seen alongside hepatic vein systolic flow reversal. There was also a D-shaped interventricular septum during diastole, suggestive of volume overload seen in the parasternal short-axis window. Cardiovascular magnetic resonance imaging was performed, ruling out changes suggestive of arrhythmogenic cardiomyopathy but corroborating findings suggestive of severe TR with a dilated RA (RA area of 51.0 cm^2^ [normal <23.0 cm^2^]; Figure 2, Video 5). The right ventricle was also dilated (RV end-systolic volume index of 129.0 mL/m^2^ and RV end-diastolic volume index of 221.0 mL/m^2^) but with mildly reduced RV systolic function (RV ejection fraction of 42%). This led to further interrogation using transesophageal echocardiography (TEE).

**Figure 3:**
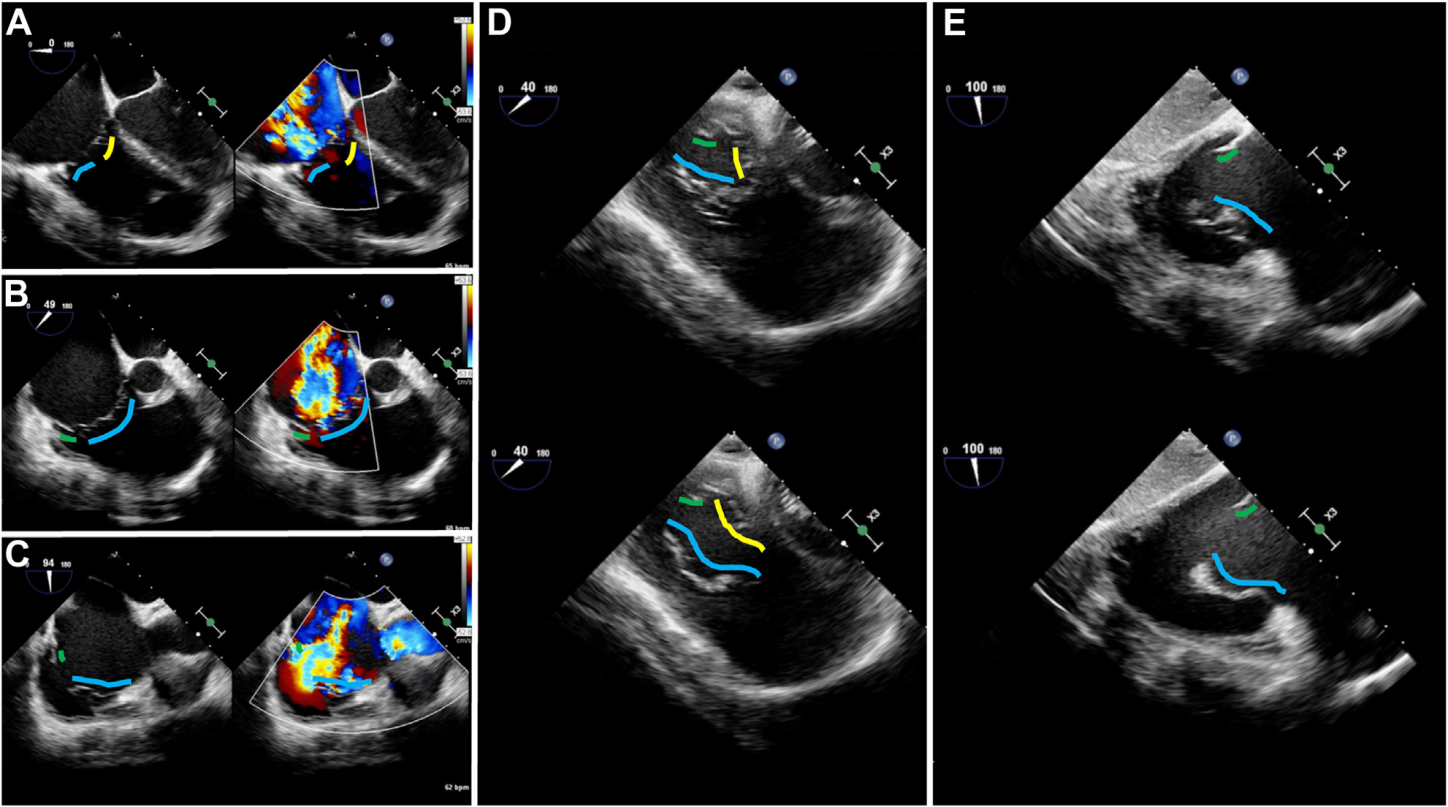
Two-dimensional TEE, midesophageal level, systolic views without (*left*) and with (*right*) color-flow Doppler in the **(A)** 4-chamber view (0°), **(B)** short-axis view (49°), and **(C)** oblique bicaval view (94°), demonstrates the morphology of the anterior (*blue*), septal (*yellow*), and posterior (*green*) TV leaflets. The posterior leaflet is tented and hypoplastic and there is severe TR. In the transgastric window, short-axis (40°) **(D)** and long-axis (100°) **(E)** views of the TV leaflets in systole (*top*) and diastole (*bottom*), the hypoplastic posterior leaflet contributing to malcoaptation is seen.

**Figure 4:**
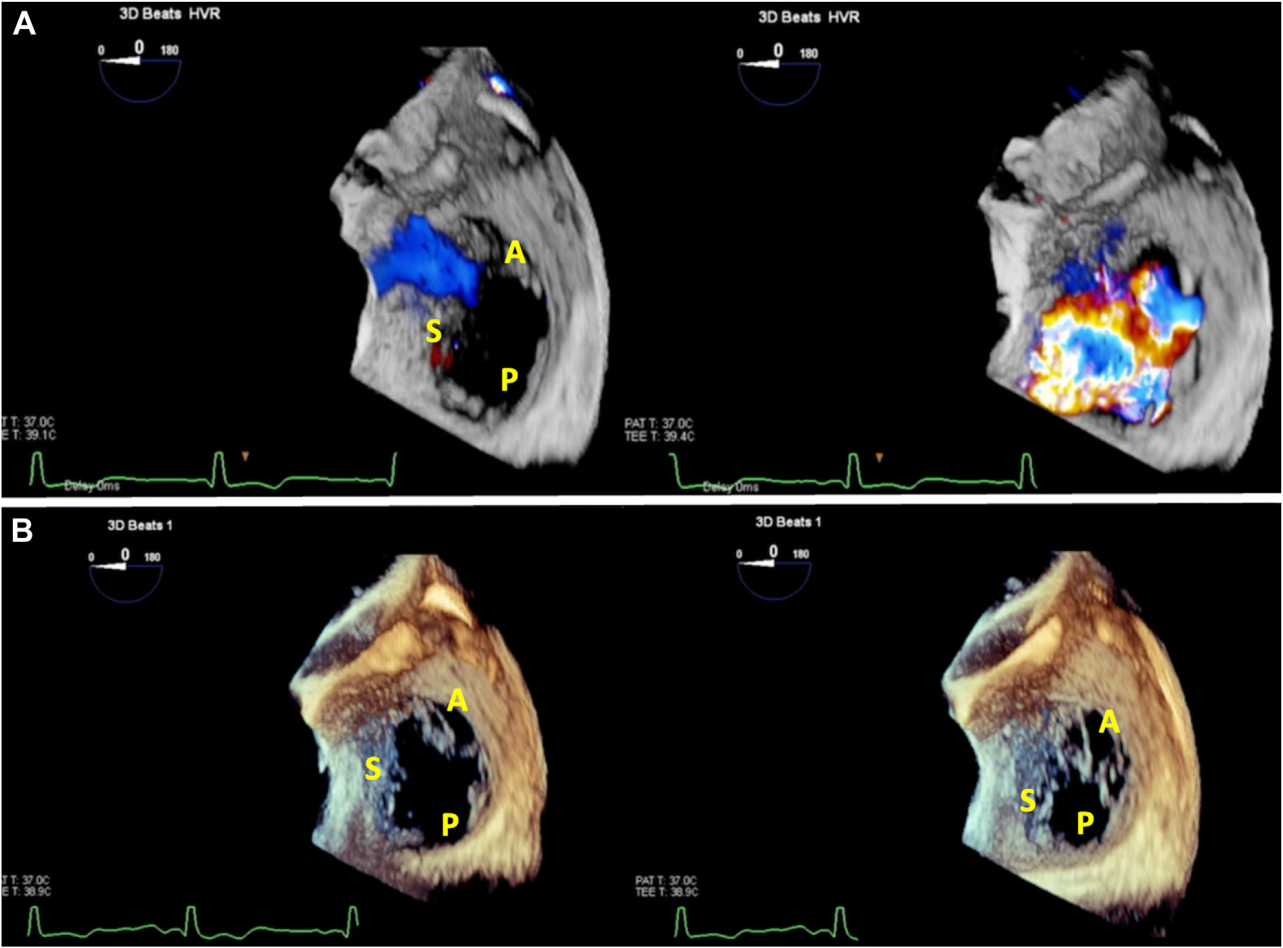
Three-dimensional TEE, single-beat, volume-rendered reconstruction, with (**A**; *top row*) and without (**B**; *bottom row*) color-flow Doppler in diastole (*left*) and systole (*right*) demonstrates the severe TR seen passing through the gap created by the hypoplastic posterior leaflet. *A*, Anterior leaflet; *P*, (hypoplastic) posterior leaflet; *S*, septal leaflet.

On TEE, there was severe TR seen originating from the posterior leaflet of the TV at the midesophageal level using various windows (0°, 40°-60°, and 90°-110°; Figure 3, Video 6). When moved to the transgastric level, using the 40° to 60° and 90° to 110° windows, it became apparent that the posterior leaflet of the TV appeared short and hypoplastic (Figure 3, Video 7). This was further confirmed using three-dimensional reconstruction, where the hypoplastic posterior leaflet alongside severe TR passing through the posterior segment gap could be seen (Figure 4, Video 8). Similar to the TTE, there was also evidence of tethering of the septal leaflet. Clinical investigations for carcinoid syndrome were all unremarkable, and the underlying partial absence of the posterior leaflet of the TV was felt to be congenital in nature. The patient was subsequently referred for TV replacement, which they underwent successfully.

## Discussion

The TV is the largest and most apically positioned valve. It is comprised of 4 major components—the fibrous annulus, leaflets, papillary muscles, and chordal attachments.^1^^,^^2^ The tricuspid leaflets are generally divided into 3—the anterior (largest), septal, and posterior (shortest circumferentially) leaflets, although there have been cases of bicuspid valves and those with more than 3 leaflets reported among healthy individuals.^1^ The posterior (or inferior) leaflet may have multiple scallops and in approximately one-tenth of cases is not clearly separated from the anterior leaflet.^1^^,^^2^ Although anatomic landmarks are rarely consistent, the commissure between the septal and posterior leaflets is usually located near the ostium of the coronary sinus. There have only been 5 cases in total of congenital absence of TV leaflets reported.^3^^,^^4^ Of these, 2 involved the anterior leaflet, with the remaining 3 involving the posterior leaflet. Differentiating complete absence, versus hypoplasia, may be important to guide direction of care, as complete absence of leaflets may preclude patients from surgical repair or procedures involving transcatheter edge-to-edge repair. Early identification is crucial since torrential TR as seen in our case can have significant hemodynamic consequences that may no longer be reversible following surgical intervention.^5^ Our patient may have had a protracted course of illness possibly due to an isolated posterior leaflet involvement (with a relatively large anterior leaflet).

Although two-dimensional TTE is often the first-line investigation performed, there remain various pitfalls especially surrounding leaflet identification. Alongside transgastric windows on two-dimensional TEE, the role of multiplanar imaging and reconstruction as well as three-dimensional reconstruction is pivotal to accurately identify the absence of leaflet segments.^2^ Multimodality imaging is also important to exclude key differentials including both infective or noninfective endocarditis, with thorough assessment of complications like abscesses, leaflet perforations, or chordae ruptures.^6^, ^7^, ^8^ Computed tomography imaging of the thorax and cardiovascular magnetic resonance imaging may also prove useful to exclude specific mimics like carcinoid disease, arrhythmogenic cardiomyopathy, or conditions linked to pulmonary hypertension (e.g., pulmonary embolism). Management of hypoplastic leaflets has largely been surgical in nature and predominantly involves valve replacement. However, with the advent of more transcatheter options, there may be potential in their use, specifically that of transcatheter prosthetic valve implantations, be it orthotopic or heterotopic valves, with little need to interact with the native TV.^6^^,^^7^ Nevertheless, data on outcomes and prognosis, with or without surgical and transcatheter interventions, remain scarce at present in view of how uncommon this condition is.

## Conclusion

Primary TR, although uncommon, is an important diagnosis not to be mistaken with “functional” TR as management between the 2 vastly differs. Early repair or replacement of the valve is paramount to avoid irreversible cardiac remodeling and its sequelae. Incorporating multimodality imaging is key in not only establishing the diagnosis and etiology but also in determining suitable management.

## Ethics Statement

The authors declare that the work described has been carried out in accordance with the following guidelines: Ethics approval was waivered by the Universiti Teknologi Mara (UiTM) Sungai Buloh Ethics Committee due to the nature of the manuscript (i.e., case report). The manuscript does not report on any animal data or tissue.

## Consent Statement

Complete written informed consent was obtained from the patient (or appropriate parent, guardian, or power of attorney) for the publication of this study and accompanying images.

## Funding Statement

The authors declare that this report did not receive any specific grant from funding agencies in the public, commercial, or not-for-profit sectors.

## Disclosure Statement

The authors report no conflict of interest.

## Supplementary Data

Video 1 Two-dimensional TTE, parasternal long-axis and parasternal short-axis views, demonstrates the normal size LV with dilated RV and septal flattening during diastole.

Video 2 Two-dimensional TTE, RV inflow view without and with color-flow Doppler, demonstrates a dilated RA and RV with severe TR. Zoomed view of the TV demonstrates the short and hypoplastic TV posterior leaflet.

Video 3 Two-dimensional TTE, parasternal short-axis view, without and with color flow Doppler, demonstrates the dilated right heart with torrential TR.

Video 4 Two-dimensional TTE, apical 4-chamber view, without and with color-flow Doppler, demonstrates the dilated right heart, the hypertrabeculated RV, and torrential TR. Zoomed view of the TV demonstrates the short, tented, and hypoplastic posterior leaflet.

Video 5 Cardiovascular magnetic resonance imaging, balanced steady-state free precession cine sequence, demonstrates the dilated RA and RV with malcoaptation of the TV and suspected severe TR.

Video 6 Two-dimensional TEE, midesophageal level, without (*left*) and with (*right*) color-flow Doppler in the 4-chamber (0°), short-axis (49°), and long-axis (94°) views demonstrates the TV leaflet morphology and severe TR. The posterior leaflet is tented and hypoplastic.

Video 7 Two-dimensional TEE, lower-esophageal (40°), and transgastric (100°), and biplane windows, demonstrates the posterior leaflet is tented and hypoplastic.

Video 8 Three-dimensional TEE, volume-rendered reconstruction, RA perspective, without and with color-flow Doppler, demonstrates severe TR passing through the gap created by the hypoplastic posterior leaflet.

## References

[1] Dahou A., Levin D., Reisman M., Hahn R.T. (2019). Anatomy and physiology of the tricuspid valve. JACC Cardiovasc Imaging.

[2] Hahn R.T. (2016). State-of-the-Art Review of echocardiographic imaging in the evaluation and treatment of functional tricuspid regurgitation. Circ Cardiovasc Imaging.

[3] Komoda T., Stamm C., Fleck E., Hetzer R. (2012). Absence of posterior tricuspid valve leaflet and valve reconstruction. Interact Cardiovasc Thorac Surg.

[4] Amhaz H., Kretzer A., Podgoreanu M., Glower D., Nicoara A. (2017). Tricuspid regurgitation due to absent tricuspid valve leaflet: utility of three-dimensional echocardiography. Anesth Analg.

[5] Prihadi E.A., Delgado V., Leon M.B., Enriquez-Sarano M., Topilsky Y., Bax J.J. (2019). Morphologic types of tricuspid regurgitation: characteristics and prognostic implications. JACC Cardiovasc Imaging.

[6] Hahn R.T., Nabauer M., Zuber M., Nazif T.M., Hausleiter J., Taramasso M. (2019). Intraprocedural imaging of transcatheter tricuspid valve interventions. JACC Cardiovasc Imaging.

[7] Khalique O.K., Cavalcante J.L., Shah D., Guta A.C., Zhan Y., Piazza N. (2019). Multimodality imaging of the tricuspid valve and right heart anatomy. JACC Cardiovasc Imaging.

[8] Hahn R.T., Thomas J.D., Khalique O.K., Cavalcante J.L., Praz F., Zoghbi W.A. (2019). Imaging assessment of tricuspid regurgitation severity. JACC Cardiovasc Imaging.

